# Assessment of Eating Behavior and Genetic Risk Factors for Metabolic Syndrome

**DOI:** 10.3390/jcm15020739

**Published:** 2026-01-16

**Authors:** Ainur Turmanbayeva, Karlygash Sadykova, Gulnaz Nuskabayeva, Ainash Oshibayeva, Ugilzhan Tatykayeva, Yusuf Ozkul, Dinara Azizkhojayeva, Dilbar Aidarbekova, Dinara Nemetova, Dana Kaldarkhan, Bibigul Tastemirova, Kanatzhan Kemelbekov

**Affiliations:** 1 Faculty of Medicine, Khoja Akhmet Yassawi International Kazakh-Turkish University, Turkestan 160000, Kazakhstan; ainur.turmanbaeva@ayu.edu.kz (A.T.); karlygash.sadykova@ayu.edu.kz (K.S.);; 2 Department of Medical Genetics, School of Medicine, Erciyes University, Kayseri 38039, Turkey; 3 Department of Pediatrics-1, South Kazakhstan Medical Academy, Shymkent 160019, Kazakhstan

**Keywords:** metabolic syndrome, eating behavior, ADIPOQ rs266729, MC4R rs17782313, gene polymorphisms

## Abstract

**Background**: Metabolic syndrome (MetS) is influenced by behavioral and genetic factors, yet evidence on eating behavior patterns and related genetic polymorphisms in Central Asian populations remains limited. Aim: The aim of this study was to assess eating behaviors among adults with and without MetS and evaluate their associations with clinical indicators and ADIPOQ rs266729 and MC4R rs17782313 variants. **Methods**: A cross-sectional study of 200 adults (115 non-MetS, 85 MetS) was conducted using Dutch Eating Behavior Questionnaire (DEBQ), standardized clinical measurements, and PCR-RFLP genotyping. **Results**: Participants with MetS were older than non-MetS adults (52 vs. 47 years; *p* = 0.004) and had substantially higher systolic blood pressure (126 vs. 114 mmHg; *p* < 0.001), diastolic blood pressure (83 vs. 74 mmHg; *p* < 0.001), and BMI (32.2 vs. 25.9 kg/m^2^; *p* < 0.001). Waist circumference, hip circumference, triglycerides, total cholesterol, and LDL were also significantly higher, while HDL was lower (1.13 ± 0.40 vs. 1.58 ± 1.50 mmol/L; *p* = 0.008). DEBQ restrained, emotional, and external eating scores showed no differences between groups (all *p* > 0.05). Eating behavior distribution was similar (*p* = 0.291). ADIPOQ genotypes (CC/CG/GG) did not differ by MetS status (*p* = 0.227), nor did MC4R variants (*p* = 0.679). Among MetS participants, clinical indicators did not vary across eating behavior categories, and no associations were observed between eating behavior and either polymorphism. **Conclusions**: Despite clear clinical and metabolic differences between MetS and non-MetS groups, neither eating behavior patterns nor ADIPOQ and MC4R variants were associated with metabolic measures among MetS group.

## 1. Introduction

Metabolic syndrome (MetS) consists of multiple interconnected metabolic problems, which include central obesity, dyslipidemia, hypertension, and insulin resistance, and it serves as a primary risk factor for developing type 2 diabetes and cardiovascular disease. Research indicates that lifestyle and environmental factors contribute to the development of MetS [1], but scientists also recognize that behavioral and genetic factors influence how individuals respond to MetS risk factors [2]. Research has started to focus on eating behavior patterns because they help scientists understand how psychological factors and behavioral responses affect food consumption, weight management, and metabolic health.

The Dutch Eating Behavior Questionnaire (DEBQ) is a proven assessment tool that identifies three main eating behavior patterns: restrictive eating, emotional eating, and external eating [3]. People who practice restrained eating purposefully restrict their food consumption to achieve weight management. The research indicates that restrained eating leads to higher BMI levels in obese adults and adolescents but fails to stop their weight from increasing [4,5]. People who engage in emotional eating consume food because they experience negative emotions, while external eaters react strongly to environmental food stimuli. External eating leads to a higher BMI because people experience stronger food cravings and heightened sensitivity to environmental triggers [6]. The predictive power of emotional eating for food consumption and body mass index remains unclear because it does not effectively identify obesity risk [4,5].

The genetic elements that control appetite and energy metabolism create differences between individuals in their eating behaviors and metabolic outcomes. The ADIPOQ gene, which encodes adiponectin, exhibits polymorphisms associated with the development of obesity, metabolic disorders, and disturbances in appetite control [7]. The rs266729 (−11377C > G) polymorphism reduces adiponectin production, which may affect how people develop emotional and external eating behaviors [8]. The MC4R gene region contains polymorphisms, including rs17782313 (C/T), which are among the most potent known genetic factors affecting obesity development and appetite control [9]. The MC4R gene functions as a key regulator of hypothalamic appetite control, and its genetic variants have been shown to increase hunger, decrease satiety, and enhance food responses to stress and environmental food stimuli. The behavioral and genetic factors that affect eating regulation need further study because they interact differently in people with metabolic syndrome who show increased clinical symptoms.

The research aims to investigate eating behaviors in MetS patients using the DEBQ, examine their relationships with medical and metabolic indicators, and assess their associations with ADIPOQ and MC4R gene variations. The research will show how these genetic variations affect metabolic indicators in this specific population. The research findings will help scientists understand the behavioral and genetic factors that contribute to metabolic syndrome, enabling them to create individualized treatment plans for lifestyle and nutritional interventions.

## 2. Materials and Methods

### 2.1. Study Design and Participants

The research study included adult participants aged 18 or older, recruited from two locations: community health centers in Turkistan City and the Clinics of Khoja Akhmet Yassawi International Turkish-Kazakh University. The clinical assessment of all participants was conducted by professional medical staff. The study used established clinical standards to identify participants with metabolic syndrome, with classification based on their metabolic health status. The study excluded participants who had active psychiatric conditions, chronic neurological diseases, severe acute illnesses, pregnancy, or any condition that would stop them from finishing the questionnaire or giving accurate answers. The laboratory received peripheral venous blood samples, processed them through sterile procedures, and placed them in EDTA tubes, which were stored at −20 °C until genetic tests were performed.

### 2.2. Ethical Approval

The Institutional Research Ethics Committee of Khoja Akhmet Yassawi International Turkish-Kazakh University approved the study protocol under protocol № 30 on 30 May 2024. The research adhered to all ethical standards outlined in the Declaration of Helsinki. The research team obtained written consent from all participants before they began participating in the study.

### 2.3. Assessment of Eating Behavior

The Dutch Eating Behavior Questionnaire (DEBQ), validated for Dutch populations, assessed eating behavior patterns through three dimensions: restrained eating, emotional eating, and external eating. The questionnaire contains 33 items, which participants rate on a 5-point Likert scale to obtain subscale scores through established scoring methods. The research team conducted a comprehensive data review before beginning their analysis. The researchers analyzed DEBQ scores as continuous data to examine eating behavior patterns in patients with metabolic syndrome and their relationships with medical and metabolic markers.

### 2.4. Genotyping Procedures

The process of genomic DNA extraction from peripheral venous blood involved standard phenol–chloroform extraction methods. The research focused on two specific single-nucleotide polymorphisms, ADIPOQ rs266729 (−11377C > G) and MC4R rs17782313 (C/T), because they are linked to appetite control, obesity, and metabolic health risks. The PCR reaction mixture contained 25 μL of nuclease-free water, 10× PCR buffer with Mg^2+^ and dNTP mix (2.5 mM each), forward and reverse primers (10 pmol/μL), Taq DNA polymerase, and 200 ng of genomic DNA template. The thermal cycling program required individual optimization for each polymorphism based on primer melting temperatures and predicted amplicon lengths. The PCR protocol for rs266729 started with 5 min at 95 °C, followed by 40 cycles of 30 s at 95 °C for denaturation, 30 s at 54–60 °C for annealing, and 45 s at 72 °C for extension, before ending with 5 min at 72 °C. The PCR protocol for rs17782313 started with 5 min at 95 °C, followed by 40 cycles of 30 s at 95 °C for denaturation, 30 s at 58–62 °C for annealing, and 1 min at 72 °C for extension, before finishing with 5 min at 72 °C. The PCR products were electrophoresed on 2% agarose gels to confirm successful amplification.

The RFLP method enabled researchers to identify different genotypes. The 30 μL digestion reaction contained the specific restriction enzyme and buffer for each target polymorphism, together with nuclease-free water and 10 μL of PCR product. The samples underwent incubation at 37 °C for 16 h. The agarose gel separation of digestion products, with ethidium bromide staining, allowed researchers to detect specific band patterns indicative of alleles. Two laboratory specialists independently verified all genotype results. The research team performed re-genotyping on 10% of randomly chosen samples to verify both quality control and genotyping precision.

### 2.5. Clinical and Metabolic Measurements

The research team obtained standardized anthropometric data by measuring height and weight, waist circumference, and body mass index (BMI). The automated sphygmomanometer measured blood pressure after participants rested for at least 5 min. The university clinical laboratory performed automated biochemical analyzer tests on fasting blood samples to determine glucose, insulin, triglyceride, total cholesterol, HDL cholesterol, and LDL cholesterol levels. The researchers used established mathematical formulas to assess Homeostasis Model Assessment of Insulin Resistance (HOMA-IR) values. The research team evaluated how eating behavior patterns and genetic variations relate to metabolic syndrome characteristics through these clinical and metabolic measurements.

### 2.6. Statistical Analysis

Data analyses were conducted using Stata version 17.0 (StataCorp LLC, College Station, TX, USA). Continuous variables were described using means and standard deviations, whereas categorical variables were presented as counts and percentages. Group differences in continuous measures were assessed with Student’s *t*-test, and categorical comparisons were examined using chi-square tests. When assumptions for parametric testing were not met, the Mann–Whitney U test and Fisher’s exact test were applied. Comparisons of continuous variables across the three eating behavior categories were performed using a one-way ANOVA or the Kruskal–Wallis test, depending on data normality. A two-sided *p*-value of <0.05 was considered statistically significant.

## 3. Results

The research included 200 adult participants, comprising 115 (57%) non-MetS participants and 85 (43%) participants who fulfilled MS criteria (Table 1). The average age was 49 ± 11 years, but MetS group members were older than non-MS group members (52 ± 11 years vs. 47 ± 11 years; *p* = 0.004). The majority of participants were female (75%), Kazakh (80%), and married (85%). The participants showed similar patterns of smoking and alcohol consumption, and there were no significant differences between groups.

**Table 1:** Socio-demographic and clinical characteristics of participants with and without metabolic syndrome (*n* = 200).

	Total	Metabolic Syndrome		*p*-Value
		No (*n* = 115; 57%)	Yes (*n* = 85; 43%)	
*Socio-demographic characteristics*				
Age, year (mean ± SD)	49 (11)	47 (11)	52 (11)	0.004
Gender, *n* (%)				0.804
Female	150 (75)	87 (76)	63 (74)	
Male	50 (25)	28 (24)	22 (26)	
Ethnicity, *n* (%)				0.721
Kazakh	160 (80)	93 (81)	67 (79)	
Others	40 (20)	22 (19)	18 (21)	
Marital status, *n* (%)				0.099
Single	31 (15)	22 (19)	9 (11)	
Married	169 (85)	93 (81)	76 (89)	
Education level, *n* (%)				0.707
Low	33 (17)	18 (16)	15 (18)	
High	167 (83)	97 (84)	70 (83)	
Smoking, *n* (%)				0.148
No	177 (89)	105 (91)	72 (85)	
Yes	23 (11)	10 (9)	13 (15)	
Alcohol drinking, *n* (%)				0.160
No	162 (81)	97 (84)	65 (76)	
Yes	38 (19)	18 (16)	20 (2)	
*Clinical characteristics*				
Systolic blood pressure, mmHg (mean ± SD)	119 (16)	114 (14)	126 (15)	<0.001
Diastolic blood pressure, mmHg (mean ± SD)	78 (11)	74 (10)	83 (10)	<0.001
Heart rate, bpm (mean ± SD)	77 (8)	76 (8)	79 (8)	0.002
Waist circumference, cm (mean ± SD)	94 (15)	87 (13)	103 (12)	<0.001
Hip circumference, cm (mean ± SD)	105 (12)	101 (11)	111 (11)	<0.001
BMI, kg/m^2^ (mean ± SD)	28.6 (5.6)	25.9 (4.1)	32.2 (5.4)	<0.001
Cholesterol, mmol/L (mean ± SD)	4.98 (0.9)	4.81 (0.9)	5.20 (0.9)	0.006
Triglycerides, mmol/L (mean ± SD)	1.57 (0.8)	1.26 (0.6)	1.99 (0.9)	<0.001
HDL, mmol/L (mean ± SD)	1.39 (1.2)	1.58 (1.5)	1.13 (0.4)	0.008
LDL, mmol/L (mean ± SD)	2.93 (0.8)	2.83 (0.7)	3.06 (0.9)	0.038
Glucose, mmol/L (mean ± SD)	5.23 (0.79)	5.03 (0.5)	5.49 (1.02)	<0.001
Fasting glucose, mmol/L (mean ± SD)	5.44 (1.01)	5.17 (0.9)	5.81 (1.05)	<0.001
Glucose after 2 h, mmol/L (mean ± SD)	5.96 (1.3)	5.65 (0.9)	6.38 (1.6)	<0.001
HbA1c, % (mean ± SD)	5.69 (0.9)	5.49 (0.7)	5.99 (1.2)	<0.001
Taking antihypertensive drugs, *n* (%)				<0.001
No	148 (74)	100 (87)	48 (56)	
Yes	52 (26)	15 (13)	37 (44)	
Taking antidiabetic drugs, *n* (%)				0.011
No	192 (96)	114 (99)	78 (92)	
Yes	8 (4)	1 (1)	7 (8)	
Taking lipid-lowering drugs, *n* (%)				0.001
No	192 (96)	115 (100)	77 (91)	
Yes	8 (4)	0	8 (9)	

The DEBQ scores did not differ significantly between participants with and without metabolic syndrome (Table 2). The two groups showed identical scores for restrained eating. The scores for emotional and external eating showed no differences between groups (*p* = 0.487 and *p* = 0.447, respectively). The distribution of eating behavior did not differ by MetS status (*p* = 0.291). The ADIPOQ CC, CG, and GG genotypes occurred at the same frequency in both groups (*p* = 0.227) (Table 2). The MC4R gene variants showed no difference between groups (*p* = 0.679), with TC and TT being the most prevalent variants at 45% and 31% respectively.

**Table 2:** Eating behavior characteristics and gene polymorphisms among participants with and without MetS (*n* = 200).

	Total	Metabolic Syndrome		*p*-Value
		No (*n* = 115; 57%)	Yes (*n* = 85; 43%)	
*DEBQ items*				
Restrained eating, (mean ± SD)	2.27 (0.9)	2.27 (0.9)	2.27 (0.9)	0.999
Emotional eating, (mean ± SD)	1.95 (0.8)	1.99 (0.9)	1.90 (0.8)	0.487
External eating, (mean ± SD)	2.55 (1.8)	1.46 (0.8)	2.67 (2.7)	0.447
Dominant eating type, *n* (%)				0.291
Restrained eating	67 (33)	37 (32)	30 (35)	
Emotional eating	41 (21)	28 (24)	13 (15)	
External eating	92 (46)	50 (44)	42 (50)	
*Gene polymorphisms*				
*ADIPOQ*, *n* (%)				0.227
CC	98 (49)	60 (52)	38 (45)	
CG	76 (38)	38 (33)	38 (45)	
GG	26 (13)	17 (15)	9 (10)	
*MC4R*, *n* (%)				0.679
CC	49 (24)	30 (26)	19 (22)	
TC	89 (45)	51 (45)	37 (44)	
TT	62 (31)	33 (29)	29 (34)	

The study revealed significant differences in participants’ clinical characteristics across study groups. Participants with MetS had higher blood pressure readings than those without MetS: 126 ± 15 mmHg for systolic and 83 ± 10 mmHg for diastolic; both differences were statistically significant. Participants with MetS had higher waist circumference, hip circumference, and BMI than the non-MetS group (*p* < 0.001 for all). The metabolic parameters between groups also showed significant differences. Participants with MetS showed elevated levels of total cholesterol (5.20 ± 0.90 mmol/L), triglycerides (1.99 ± 0.90 mmol/L), and LDL cholesterol (3.06 ± 0.90 mmol/L) compared to the non-MetS group (*p* = 0.006, *p* < 0.001, and *p* = 0.038, respectively.

Participants’ medication use patterns reflected the extent of their metabolic syndrome. Participants with MetS were more likely to require antihypertensive medication, antidiabetic drugs, and lipid-lowering medications than non-MetS participants.

The association between eating behavior types, clinical characteristics, and gene polymorphisms among participants with metabolic syndrome is presented in Table 3. Participants with metabolic syndrome showed no association between their eating behavior types (restrained eating, emotional eating, external eating) and their clinical outcomes. The mean systolic blood pressure measurements of external eaters (117 ± 17 mmHg) were slightly higher than those of restrained eaters (112 ± 11 mmHg) and emotional eaters (111 ± 13 mmHg). Still, the difference was not statistically significant (*p* = 0.108). The study results showed no differences in hypertension prevalence between groups (*p* = 0.400).

The study found no association between eating behavior patterns and variations in the ADIPOQ and MC4R gene sequences. The ADIPOQ genotypes (CC, CG, GG) followed the same pattern across all three eating behavior groups (*p* = 0.960). The MC4R genotypes showed no statistically significant differences between groups (*p* = 0.823), while the TC genotype was most frequent across all studied categories. The influence of the studied gene polymorphisms on clinical and metabolic parameters of participants with MetS is presented in Table S1.

## 4. Discussion

This study examined eating behavior patterns, clinical characteristics, and two obesity-related gene polymorphisms among adults with and without metabolic syndrome. The overall findings indicate that individuals with MetS exhibit a clearly more adverse clinical and metabolic profile, but their behavioral eating patterns and genetic variants related to appetite regulation do not differ from those of individuals without MetS. Furthermore, among participants with metabolic syndrome, neither eating behavior type nor the studied polymorphisms were associated with clinical or metabolic indicators. These findings emphasize the predominance of metabolic and physiological determinants of MetS over behavioral or single-gene factors in this population.

The significant differences in metabolic and anthropometric measures between MetS and non-MetS participants align with extensive global evidence showing that central adiposity, dyslipidemia, and elevated blood pressure are core features of metabolic syndrome [10,11]. A longitudinal twin study from Korea used the DEBQ and found some associations between eating behavior and MetS risk, but these behavioral effects were modest and varied by sex and eating behavior [12]. A review by Goldbacher et al. discusses psychological traits in relation to MetS components but highlights that physiological mechanisms, such as hypothalamic–sympathetic dysregulation, are likely central [13]. Our findings support this understanding, indicating that the physiological burden of MetS can be identified regardless of differences in self-reported eating behavior.

The absence of differences in DEBQ scores between participants with and without MetS contrasts with some studies reporting that higher emotional or external eating is linked to weight gain or obesity. Research from Algeria [14], Spain [15], and Turkey [16] has shown that emotional and external eating may predict increased caloric intake and higher BMI. However, other studies have reported weak or inconsistent associations between DEBQ scores and metabolic outcomes, particularly in clinical populations already exhibiting chronic metabolic disorders. One possible explanation is that once individuals develop MetS, biological drivers such as insulin resistance, leptin resistance, and chronic inflammation may overshadow behavioral influences on eating. Koide et al. found no significant associations between emotional or restrained DEBQ scores and change in HbA1c over 12 months [17]. Another study shows some associations between external eating and higher HbA1c; however, it also documents that these relationships vary by sex, obesity status, and diabetes duration [18]. In addition, individuals with chronic conditions may engage in more conscious or regulated eating due to medical advice, leading to scores that resemble those of healthier individuals. This reinforces the importance of considering compensatory behavioral changes in MetS populations when interpreting DEBQ results.

The Dutch Eating Behavior Questionnaire was used to evaluate stable eating patterns, including cognitive restraint, emotional responsiveness, and food cue sensitivity, rather than measuring actual food consumption or specific diet components. The DEBQ measures eating behavior traits, but it does not show how many calories people eat, what nutrients they consume, or when they eat their meals [19]. The distinction becomes essential when studying clinical patients, because their metabolic results derive from physical body system issues rather than their self-reported behavioral symptoms. In addition, people who receive a Metabolic Syndrome diagnosis might develop new eating patterns, including better food control and purposeful changes in their eating habits, after receiving medical advice [20]. The eating behavior scores between MetS patients and non-MetS patients may appear similar because MetS patients learn to control their eating through dietary restraint and purposeful dietary changes. The established metabolic syndrome condition creates physiological changes that make it difficult to detect how behavioral characteristics and individual gene variations affect patients in studies that measure participants at one point in time [21].

The lack of associations between eating behavior types and clinical indicators among participants with MetS is consistent with the literature, which suggests that behavioral traits alone rarely predict blood pressure, lipid levels, or glycemic status once metabolic dysfunction is established [22]. Studies in cardiometabolic cohorts have shown that physiological mechanisms, including visceral fat accumulation, altered adipokine secretion, and sympathetic overactivation, drive most metabolic abnormalities independently of cognitive restraint, emotional triggers, or environmental food responsiveness [23,24]. Thus, our findings support the notion that while eating behavior contributes to long-term weight regulation, its influence may diminish once MetS is clinically manifested.

Regarding gene polymorphisms, prior research has linked the ADIPOQ rs266729 polymorphism to reduced adiponectin levels, insulin resistance, and increased obesity susceptibility [25,26]. Several studies in Southeast Asian and Middle Eastern populations have shown positive associations [26,27]. In contrast, European and Central Asian studies often report null findings for differences in anthropometric indicators among carriers of different ADIPOQ genotypes [28,29]. The lack of association in our sample suggests that rs266729 may exert a weak effect in this population, or that lifestyle and environmental factors may play a more dominant role in metabolic risk than single-gene polymorphisms. However, in prior research among Kazakh adults, the rs266729 variant of the ADIPOQ gene was significantly associated with both type 2 diabetes and obesity [29]. Taken together, these mixed findings suggest that the metabolic impact of ADIPOQ variation may be population-specific and may depend on broader genetic background, interactions with dietary patterns, or the stage of metabolic disease at the time of assessment.

Likewise, the MC4R rs17782313 variant is among the most widely studied genetic markers for appetite regulation and obesity risk [30]. Some research in European and Middle East Asian cohorts has linked this variant to increased hunger, reduced satiety, and higher scores on measures of external or emotional eating [9,31]. However, other studies have found no association with eating behavior or metabolic syndrome [32,33]. The lack of association in our study may reflect population-specific genetic architecture, gene–environment interactions, or the possibility that DEBQ may not fully capture the subtle appetite-regulation differences influenced by MC4R variants.

Minimal findings from this research suggest that the effects of genetics or behavior may be too slight to detect with the current sample size. While our study was powered to identify moderate between-group differences, smaller effect sizes, particularly those typical of single-gene polymorphisms or behavioral traits, may have gone undetected. The populations in Central Asia may also have different genetic makeups compared to those in Europe and East Asia, possibly with varying patterns of alleles, genetic linkages, and gene–environment interactions [34,35].

The Dutch Eating Behaviour Questionnaire has been validated across many ethnic groups; however, its statistical properties have not been extensively tested in Central Asian communities. Cross-cultural variations in how people express emotions, their eating habits, and customs can lead to different reactions to questions about hunger and food desires. In a large population-based cross-sectional study of Kazakh adults, investigators applied three widely used diagnostic criteria for metabolic syndrome (ATP III, IDF, and JIS) [36]. They demonstrated that the prevalence of MetS among Kazakhs exceeded national estimates for China and lay between levels observed in Euro-American and East Asian populations. The study’s findings revealed that waist circumference cut-offs used in China may not be the best indicators for Kazakhstan [36]. These findings underscore the importance of population- and culture-specific adaptation of diagnostic tools and support the notion that behavioral assessment instruments, such as the DEBQ, may also require cultural adjustment and validation to capture eating behavior patterns in Central Asian populations accurately.

Research studies on ADIPOQ and MC4R gene variations and their link to obesity and metabolic syndrome have produced conflicting findings, which depend on the studied population and their geographic location. Studies across European populations have shown that MC4R rs17782313 increases obesity risk by associating with higher BMI [37]. Still, it shows only a limited association with MetS components, which become less significant after adjustment for lifestyle factors [38]. Research on African populations shows limited results, indicating that their allele frequencies vary widely, while their genetic relationships are either absent or weak because their genetic makeup differs from that of other populations, and their environments play a significant role [39,40]. Studies conducted in Asian populations across East and Southeast Asia have shown that ADIPOQ and MC4R gene variants are associated with obesity, insulin resistance, and MetS-related characteristics [41]. Still, the research findings show minimal and context-dependent effects. Data from Central Asia are scarce, and existing studies report mixed results, underscoring the lack of region-specific genetic evidence. Within this context, the present findings contribute novel data from a Central Asian population and suggest that the influence of ADIPOQ and MC4R variants on metabolic syndrome may be population-specific and modified by local genetic background, lifestyle, and environmental exposures.

This study has several important strengths. It employed a comprehensive multidimensional approach by integrating anthropometric, metabolic, behavioral, and genetic data, enabling a holistic evaluation of factors associated with metabolic syndrome. The framework allows scientists to understand how metabolic syndrome develops through physiological dysregulation that becomes more pronounced at later stages. However, behavioral factors and genetic elements tend to influence the condition during its initial development. The DEBQ assessment of eating behavior shows a better ability to predict the development of metabolic risk over time than it does to study existing diseases through cross-sectional research. All clinical measurements were obtained by trained medical personnel using standardized procedures, and eating behavior was assessed with the validated Dutch Eating Behavior Questionnaire, ensuring methodological rigor. PCR-based genotyping-RFLP was conducted with strict quality control, thereby enhancing the reliability of the genetic findings. Additionally, the study contributes valuable evidence from an understudied Central Asian population, offering insights that are often missing from global metabolic and genetic research.

However, the study also has limitations that should be considered when interpreting the results. Its cross-sectional design restricts causal inference and prevents establishing temporal relationships between eating behaviors, genetic variants, and metabolic outcomes. The sample size, although adequate for preliminary analysis, may have been too small to detect subtle gene-behavior or gene–environment interactions. In addition, although previous studies have reported sex- and age-specific associations between eating behaviors and metabolic risk, the limited sample size within subgroups resulted in insufficient statistical power, and stratified models were therefore not performed. The study included participants from a single geographic area and relied on self-reported eating habits, which may lead to reporting bias. Only two polymorphisms were examined, despite the highly polygenic nature of metabolic syndrome, and the lack of dietary intake, physical activity, and biomarker data, such as adiponectin levels, limits the ability to contextualize genetic findings and fully explore mechanisms linking behavior, genotype, and metabolic health. This research found that the rs266729 variant leads to decreased adiponectin production, but did not measure blood adiponectin levels. Further studies that measure adiponectin levels as an intermediate outcome could help understand how ADIPOQ gene variations affect metabolic health and food consumption patterns.

## 5. Conclusions

This study contributes to the growing body of evidence emphasizing that metabolic syndrome is a multifactorial condition shaped predominantly by physiological and metabolic disturbances. While eating behavior and genetic predisposition remain relevant to obesity risk, they did not show significant associations with metabolic syndrome or its clinical features in this study population. These findings underscore the need for integrated approaches that combine clinical monitoring, metabolic profiling, and targeted lifestyle interventions, rather than relying solely on behavioral or genetic predictors.

## Figures and Tables

**Table 3:** Relation between eating types and clinical characteristics of patients with MetS (*n* = 115).

	Retrained Eating (*n* = 37; 32%)	Emotional Eating (*n* = 28; 24%)	External Eating (*n =* 50; 44%)	*p*-Value
*Clinical characteristics*				
Waist circumference, cm (mean ± SD)	89 (12)	82 (13)	88 (14)	0.075
Triglycerides, mmol/L (mean ± SD)	1.31 (0.7)	1.28 (0.8)	1.22 (0.5)	0.779
Glucose, mmol/L (mean ± SD)	5.05 (0.4)	5.09 (0.5)	4.99 (0.5)	0.722
HDL, mmol/L (mean ± SD)	1.36 (0.5)	1.83 (2.3)	1.59 (1.5)	0.451
LDL, (mmol/L mean ± SD)	2.79 (0.6)	2.76 (0.7)	2.91 (0.7)	0.633
Systolic blood pressure, mmHg (mean ± SD)	112 (11)	111 (13)	117 (17)	0.108
Diastolic blood pressure, mmHg (mean ± SD)	73 (8)	73 (9)	76 (12)	0.244
Hypertension, *n* (%)				0.400
No	30 (81)	19 (68)	35 (70)	
Yes	7 (19)	9 (32)	15 (30)	
*Gene polymorphisms*				
*ADIPOQ*, *n* (%)				0.960
CC	20 (54)	15 (54)	25 (50)	
CG	12 (32)	8 (28)	18 (36)	
GG	5 (14)	5 (18)	7 (14)	
*MC4R, n* (%)				0.823
CC	9 (24)	6 (21)	15 (30)	
TC	19 (52)	13 (47)	20 (40)	
TT	9 (24)	9 (32)	15 (30)	

## Data Availability

The data presented in this study are available on request from the corresponding author due to privacy reasons.

## Supplementary Materials

The following supporting information can be downloaded at: https://www.mdpi.com/article/10.3390/jcm15020739/s1, Table S1: The influence of the studied gene polymorphisms on clinical and metabolic parameters of participants with MetS.
