# Supplementary material for: Assessment of Eating Behavior and Genetic Risk Factors for Metabolic Syndrome
Source: J Clin Med. 2026 Jan 16;15(2):739. doi: 10.3390/jcm15020739 (PMC12842093; doi:10.3390/jcm15020739)
Supplement: Supplementary file 1 [file jcm-15-00739-s001.zip › jcm-4080751-supplementary.pdf]

Table S1. The influence of the studied gene polymorphisms on clinical and metabolic parameters of participants with MetS.

	ADIPOQ gene			p-value	*MC4R* gene			p-value
	**CC**	**CG**	**GG**		**CC**	**TC**	**TT**	
Waist circumference, cm (mean±SD)	85 (12)	88 (14)	91 (16)	0.238	85 (12)	85 (13)	92 (13)	0.051
Triglycerides, mmol/L (mean±SD)	1.16 (0.6)	1.42 (0.8)	1.27 (0.4)	0.132	1.26 (0.8)	1.29 (0.7)	1.22 (0.4)	0.906
Glucose, mmol/L (mean±SD)	5.05 (0.5)	5.02 (0.4)	5.01 (0.5)	0.947	5.02 (0.5)	5.04 (0.5)	5.04 (0.4)	0.985
HDL, mmol/L (mean±SD)	1.43 (0.5)	1.95 (2.5)	1.24 (0.4)	0.152	1.70 (2.2)	1.57 (1.4)	1.46 (0.4)	0.939
LDL, mmol/L (mean±SD)	2.76 (0.7)	2.95 (0.7)	2.81 (0.6)	0.425	2.86 (0.7)	2.81 (0.7)	2.84 (0.7)	0.965
Systolic blood pressure, mmHg (mean±SD)	114 (12)	114 (17)	114 (17)	0.999	111 (14)	113 (11)	117 (19)	0.418
Diastolic blood pressure, mmHg (mean±SD)	75 (9)	74 (12)	73 (11)	0.752	73 (10)	74 (10)	76 (11)	0.399
Hypertension, n (%)				0.904				0.773
No	52 (53)	38 (50)	14 (54)		27 (55)	47 (53)	30 (48)	
Yes	46 (47)	38 (50)	12 (46)		22 (45)	42 (47)	32 (52)	
